# Briarane-Related Diterpenoids from Octocoral *Briareum stechei*

**DOI:** 10.3390/molecules26226861

**Published:** 2021-11-13

**Authors:** Thanh-Hao Huynh, Choo-Aun Neoh, Yu-Chi Tsai, Zhi-Kang Yao, Li-Guo Zheng, Pin-Chang Huang, Zhi-Hong Wen, Jih-Jung Chen, Yu-Jen Wu, Ping-Jyun Sung

**Affiliations:** 1 Department of Marine Biotechnology and Resources, National Sun Yat-sen University, Kaohsiung 804201, Taiwan; d095020008@student.nsysu.edu.tw (T.-H.H.); akang329@vghks.gov.tw (Z.-K.Y.); wzh@mail.nsysu.edu.tw (Z.-H.W.); 2 National Museum of Marine Biology and Aquarium, Pingtung 944401, Taiwan; yuchi0713@nmmba.gov.tw (Y.-C.T.); d095620002@nsysu.edu.tw (L.-G.Z.); d095620001@nsysu.edu.tw (P.-C.H.); 3 Department of Research, Pingtung Christian Hospital, Pingtung 900026, Taiwan; 01071@ptch.org.tw; 4 Department of Orthopedics, Kaohsiung Veterans General Hospital, Kaohsiung 813414, Taiwan; 5 Institute of BioPharmaceutical Sciences, National Sun Yat-sen University, Kaohsiung 804201, Taiwan; 6 Department of Pharmacy, School of Pharmaceutical Sciences, National Yang Ming Chiao Tung University, Taipei 112304, Taiwan; jjungchen@nycu.edu.tw; 7 Department of Food Science and Nutrition, Meiho University, Pingtung 912009, Taiwan; 8 Chinese Medicine Research and Development Center, China Medical University Hospital, Taichung 404332, Taiwan; 9 Graduate Institute of Natural Products, Kaohsiung Medical University, Kaohsiung 807378, Taiwan; 10 Ph.D. Program in Pharmaceutical Biotechnology, Fu Jen Catholic University, New Taipei 242062, Taiwan

**Keywords:** *Briareum stechei*, briarane, briaexcavatolie, briastecholide, iNOS

## Abstract

A known polyoxygenated briarane, briaexcavatolide P (**1**), was isolated from a Formosan octocoral *Briareum stechei*. Moreover, the same species *B. stechei*, collected from Okinawan waters, yielded three chlorine-containing briaranes, including two new compounds, briastecholides B (**2**) and C (**3**) as well as a known analogue, briarenol R (**4**). The structures of **1**–**4** were established using spectroscopic methods. In addition, briarane **1** demonstrated anti-inflammatory activity in lipo-polysaccharide-induced RAW 264.7 mouse macrophage cells by suppressing the expression of inducible nitric oxide synthase (iNOS) protein.

## 1. Introduction

Soft corals are widely distributed marine invertebrates, particularly in the tropical Indo-Pacific Ocean, and have been proven to provide a wide range of diterpenoid derivatives featuring unusual carbon skeletons and possessing medicinal activities [1,2,3,4]. The octocoral *Briareum* Blainville, 1834 (family: Briareidae) [5,6,7,8] is worth studying among these marine invertebrates. There are four valid species, *B. cylindrum*, *B. hamrum*, *B. stechei*, and *B. violaceum*, distributed in the Indo-Pacific Ocean [8]. Moreover, diverse marine diterpenoids, such as briaranes (3,8-cyclized cembranoid) and eunicellins (2,11-cyclized cembranoid) [9,10], were obtained from these interesting potentially medicinal *Briareum* species.

**Figure 1:**
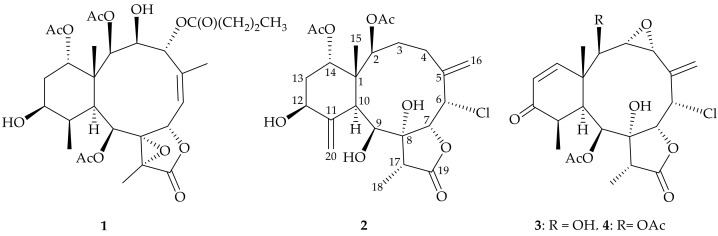
Structures of briaexcavatolide P (**1**), briastecholides B (**2**) and C (**3**), and briarenol R (**4**).

Since 1977, when the first briarane-type diterpenoid was isolated from a Caribbean octocoral, *Briareum asbestinum* [11], hundreds of briarane-type diterpenoids have been obtained from various *Briareum* spp., and the compounds of this type are only found in marine invertebrates. The briarane-type diterpenoids have been reported to exhibit several biological effects, including anti-inflammatory activity [12], cytotoxicity [13,14], and antiviral activity [13,14]. In our continuing research on natural substances from the marine invertebrates originally distributed in the tropical Indo-Pacific Ocean, two samples of the octocoral *Briareum stechei* were collected from two positions surrounded by the Kuroshio current for their interesting chemical constituents. We report on a known polyoxygenated briarane, briaexcavatolide P (**1**) [15], from a Formosan *B. stechei*, and three chlorinated briaranes, including two new metabolites, briastecholides B (**2**) and C (**3**), as well as a known analogue, briarenol R (**4**) [16] (Figure 1), from an Okinawan *B. stechei*. Isolates **1**–**4** were evaluated for their anti-inflammatory activity using the inhibition of inducible nitric oxide synthase (iNOS) in an in vitro pro-inflammatory model.

## 2. Results and Discussion

### 2.1. Structure Determination of Briaexcavatolide P from a Formosan Briareum stechei

Briarane **1** was obtained as an amorphous powder. The positive mode electrospray ionization mass spectrum (+)-ESIMS showed a peak at *m/z* 633 [M + Na]^+^ and was found to have the molecular formula C_30_H_42_O_13_ by the analysis of ^13^C and ^1^H NMR data. The result revealed that this compound had 10 degrees of unsaturation. Strong bands at 3488, 1783, and 1731 cm^−^^1^ in the IR spectrum indicated the presence of hydroxy, δ-lactone, and ester groups [17]. The ^13^C NMR and distortionless enhancement by polarization transfer (DEPT) spectra revealed that **1** had 30 carbons, including 8 methyls, 3 sp^3^ methylenes, 9 sp^3^ methines, 1 sp^2^ methine, 3 sp^3^ non-protonated carbons, and 6 sp^2^ non-protonated carbons. Therefore, **1** was identified as having four rings. It was found that the spectroscopic data of **1** were identical to those of a known briarane, briaexcavatolide P, and **1** possessed the positive optical rotation value, ${\left[\alpha \right]}_{D}^{25}$ + 223 (*c* 0.04, CHCl_3_), as that of briaexcavatolide P (${\left[\alpha \right]}_{D}^{27}$ + 167 (*c* 1.0, CHCl_3_)) [15], suggesting that **1** is briaexcavatolide P.

### 2.2. Structure Determination of Briastecholides B and C, and Briarenol R from an Okinawan Briareum stechei

Briastecholide B (**2**) was obtained as an amorphous powder. The positive mode high-resolution electrospray ionization mass spectrum (+)-HRESIMS showed sodium adduct ions at *m/z* = 523.1703 and 525.1684 with a 3:1 ratio, indicating the presence of a chlorine atom in **2**, and its molecular formula was further established as C_24_H_33_ClO_9_ (calculated considering that C_24_H_33_^35^ClO_9_ + Na and C_24_H_33_^37^ClO_9_ + Na are 523.1705 and 525.1676, respectively) (index of hydrogen deficiency, IHD = 8). The IR spectrum showed the presence of hydroxy (ν_max_ 3490 cm^−^^1^), γ-lactone (ν_max_ 1774 cm^−^^1^), and ester carbonyl (ν_max_ 1735 cm^−^^1^) functionalities. The broad peaks of ^1^H and ^13^C NMR signals were observed in the one-dimensional nuclear magnetic resonance (1D NMR) spectroscopy of **2** at 25 °C in CDCl_3_ initially; however, it was found that the NMR signals for those protons and carbons of this molecule could be assigned by the assistance of two-dimensional nuclear magnetic resonance (2D NMR) spectroscopy in cases where the NMR spectra were measured at 25 °C in acetone-*d*_6_. In the ^13^C NMR (Table 1), heteronuclear single quantum coherence (HSQC), and heteronuclear multiple bond coherence (HMBC) spectra, the presence of two exocyclic olefins were confirmed by signals of two sp^2^ methylene carbons at δ_C_ 119.1 (CH_2_-16) and 106.6 (CH_2_-20) and two non-protonated sp^2^ carbons at 155.7 (C-11) and 148.9 (C-5), and further supported by four olefinic proton signals at δ_H_ 5.56 (1H, br s, H-16a), 5.26 (1H, br s, H-16b), 5.41 (1H, br s, H-20a), and 5.19 (1H, br s, H-20b) in the ^1^H NMR spectrum (Table 1). In addition, three carbonyl resonances at δ_C_ 175.9 (C-19) as well as 171.2 and 170.7 (2 × ester carbonyls) indicated the presence of one γ-lactone and two ester groups; two acetate methyls (δ_H_ 1.89 and 1.89, each 3H × s; δ_C_ 21.5, 21.3, 2 × CH_3_) were also observed. According to the above, five double bonds contributed five IHD; thus, the remaining three degrees of unsaturation defined **2** as a tricyclic diterpenoid.

**Figure 2:**
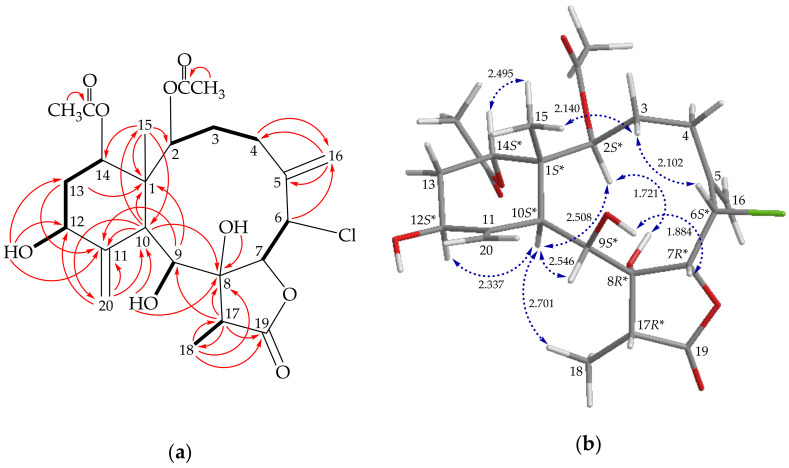
(**a**) Key COSY (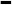), HMBC (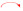), and (**b**) stereoview of **2** and calculated distances (Å) between selected protons with key NOESY (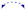) correlations.

The ^1^H-^1^H correlation spectroscopy (COSY) spin systems of H-2/H_2_-3/H_2_-4, H-6/H-7, H-12/H_2_-13/H-14, and H-17/H_3_-18 (Figure 2a) were fit to the regiochemistry of vicinal proton couplings in **2**. The cyclic network was further established by an HMBC experiment, especially by ^2^*J*- or ^3^*J*-^1^H–^13^C long-range correlations between protons and non-protonated carbons, such as H-9, H-10, H-13α/C-1; H-10/C-8; H-9, H-10, H-13α/C-11 (Figure 2a). The exocyclic olefinic double bonds attached at C-5 and C-11, respectively, were proven by the HMBC correlations between H_2_-16/C-4, C-5; H_2_-4, H-6/C-16; H_2_-20/C-10, C-11, C-12; and H-10/C-20, respectively (Figure 2a). The C-15 methyl group was sitioned at the ring junction C-1 by certification of the HMBC correlations between H_3_-15/C-1, C-2, C-10, C-14, and H-10/C-15. A hydroxy group connected to C-8 was confirmed by a critical HMBC correlation between a hydroxy proton (δ_H_ 3.76, 1H, s) and an oxygenated non-protonated carbon (δ_C_ 83.9, C-8). The other two hydroxy groups were attached to C-12 and C-9, respectively, by certification of the ^1^H–^1^H COSY correlations between OH-12 (δ_H_ 4.08, 1H, d, *J* = 6.0 Hz)/H-12 (δ_H_ 4.14, 1H, dd, *J* = 11.6, 6.0 Hz) and OH-9 (δ_H_ 5.52, 1H, d, *J* = 7.6 Hz)/H-9 (δ_H_ 4.88, 1H, d, *J* = 7.6 Hz). These findings were further confirmed by the HMBC correlations between OH-12/C-11, C-12, C-13 and OH-9/C-8, C-10. Thus, the remaining two acetoxy groups should be positioned at C-2 and C-14, respectively, as indicated by the characteristic NMR signal analysis of the oxymethine protons H-2 (δ_H_ 5.98, 1H, d, *J* = 8.8 Hz) and H-14 (δ_H_ 4.80, 1H, dd, *J* = 3.2, 3.2 Hz), although no HMBC correlation was observed between H-2 and H-14 and those acetate carbonyls.

The ^13^C NMR signal of a methine unit at δ_C_ 56.3 (CH-6) was more shielded than would be expected for an oxygenated C-atom. Furthermore, this carbon signal showed an HSQC correlation with a methine proton signal at δ_H_ 5.21, which also exhibited a COSY cross-peak with H-7 in a ^3^*J*-correlation, demonstrating the attachment of a chlorine atom at C-6. Together with the HMBC correlations between H-17/C-8, C-9, C-18, C-19 and H_3_-18/C-8, C-17, C-19, these data unambiguously established the molecular framework of **2**.

The stereochemical evaluation of **2** was approached using a nuclear Overhauser effect spectroscopy (NOESY) experiment. In the NOESY experiment (Figure 2b), H-10 correlated with H-2, H-9, H-12, and H_3_-18, respectively, indicating that these protons were situated on the same face and were assigned as α-protons; oppositely, C-15 methyl was determined as β-oriented at C-1 since H_3_-15 did not show correlation with H-10. The oxymethine proton H-14 exhibited an effect with H_3_-15 and no correlation with H-10, revealing that H-14 was β-oriented at C-14. One of the methylene protons at C-3 (δ_H_ 3.25) exhibited a correlation with H_3_-15, leading to its assignment as H-3β, while the other one was denoted as H-3α (δ_H_ 1.46). The correlation observed between H-3β and H-6 reflected the β-orientation of proton at C-6. The hydroxy protons at δ_H_ 3.76 (OH-8) and 5.52 (OH-9) displayed light correlations with H-2 and H-7, individually, setting the hydroxy groups at C-8, and the proton at C-7 were assigned as α- and β-oriented, respectively. Based on the above findings, the structure of **2** was established and the stereogenic carbons of **2** were assigned as (1S*,2S*,6S*,7R*,8R*,9S*,10S*,12S*,14S*,17R*) (Supplementary Materials, Figures S1–S9).

Briastecholide C (**3**) was isolated as an amorphous powder that showed sodium adduct ions at *m/z* 477.1284 and 479.1253 (3:1) in (+)-HRESIMS, indicating the presence of a chlorine atom, and the molecular formula was established as C_22_H_27_ClO_8_ (calculated for C_22_H_27_^35^ClO_8_ + Na, 477.1287) (IHD = 9). The IR spectrum of **3** showed the functionality signals of α,β-unsaturated ketonic group, ester carbonyl, γ-lactone, and OH stretching at 1672, 1735, 1757, and 3474 cm^−1^, respectively. Based on the ^13^C NMR data and unsaturated degree numbers, **3** was established as a tetracyclic briarane. In the ^13^C and ^1^H NMR (Table 1), HSQC, and HMBC spectra, an α,β-unsaturated ketonic group was deduced from the signals of three carbons at δ_C_ 202.5 (C-12), 123.1 (CH-13), and 155.8 (CH-14). The presence of an exocyclic olefin was confirmed by the typical signals of one sp^2^ methylene carbon at δ_C_ 118.9 (CH_2_-16) and exomethylene proton signals at δ_H_ 5.95 (1H, d, *J* = 2.8 Hz, H-16a) and 5.47 (1H, d, *J* = 2.8 Hz, H-16b). In addition, one γ-lactone, one ester, and one acetate methyl were confirmed by the NMR resonances at δ_C_ 173.8 (C-19), 169.4 (ester carbonyl), and δ_H_ 2.24 (3H, s)/δ_C_ 21.9 (acetate methyl), respectively. A disubstituted epoxy group was identified by the chemical shifts of two oxymethine carbons at δ_C_ 62.4 (CH-3) and 58.3 (CH-4) as well as their proton signals at δ_H_ 3.43 (1H, dd, *J* = 8.8, 4.0 Hz, H-3) and 3.71 (1H, d, *J* = 4.0 Hz, H-4), respectively.

**Figure 3:**
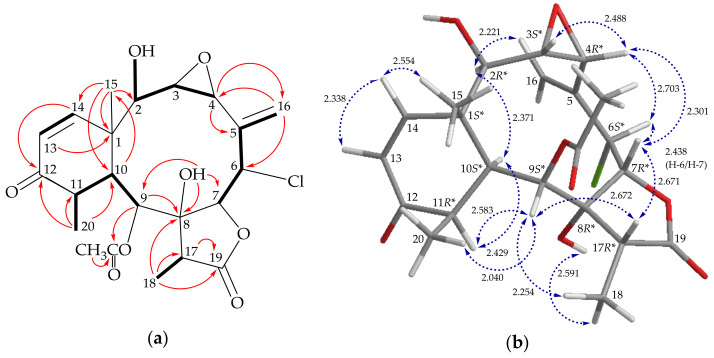
(**a**) Key COSY (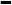), HMBC (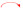), and (**b**) stereoview of **3** and calculated distances (Å) between selected protons with key NOESY (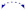) correlations.

According to the above and comparing the NMR data of **3** with those of the literature, the structure of **3** was highly similar to a known briarane, briarenol R (**4**) (Figure 1), which was originally isolated from a cultured *B. stechei* [16] and was also obtained in this study, except for a hydroxy group in **3** instead of an acetoxy group at C-2 in **4**. The HMBC and COSY correlations, as shown in Figure 3a, provided the planar structure for **3**. Both compounds **3** and **4** possessed negative values of optical rotation (**3**, ${\left[\alpha \right]}_{D}^{21}$ − 73 (*c* 0.01, CHCl_3_); **4**, ${\left[\alpha \right]}_{D}^{23}$ − 61 (*c* 0.01, CHCl_3_)), indicating that they shared similar orientations. Furthermore, in the NOESY experiment of **3**, H-2 showed a correlation with H-10, revealing the β-oriented hydroxy group at C-2 in **3**. Hence, briastecholide C (**3**) was found to be the 2-*O*-deacetyl derivative of **4** and the stereochemistry of **3** was deduced by optical rotation and NOESY analysis (Figure 3b) as (1S*,2R*,3S*,4R*,6S*,7R*,8R*,9S*,10S*,11R*, 17R*) (Supplementary Materials, Figures S10–S18).

The (+)-ESIMS mass spectra of **4** showed a pair of peaks at *m/z* 519/521 ([M + Na]^+^/[M + 2 + Na]^+^) (3:1) with a relative intensity suggestive of a chlorine atom, indicating that the molecular formula of **4** was C_24_H_29_ClO_9_. The result revealed that this compound had 10 degrees of unsaturation. Strong bands at 3452, 1783, 1742, and 1680 cm^−1^ observed in the IR spectrum confirmed the presence of hydroxy, γ-lactone, ester, and α,β-unsaturated ketonic groups. The ^13^C NMR and DEPT spectra revealed that **4** had 24 carbons, including 5 methyls, 1 sp^2^ methylene, 9 sp^3^ methines, 2 sp^2^ methines, 2 sp^3^ non-protonated carbons, and 5 sp^2^ non-protonated carbons. Therefore, **4** was identified as having four rings. It was found that the spectroscopic data of **4** were identical to those of a known briarane, briarenol R [16], and these two compounds possessed negative optical rotation values (${\left[\alpha \right]}_{D}^{23}$–61 (*c* 0.01, CHCl_3_) for **4** and ${\left[\alpha \right]}_{D}^{22}$ –55 (*c* 0.2, CHCl_3_) for briarenol R [16]); thus, compound **4** was identified as briarenol R.

Additionally, the structures of briaranes **1**–**4** were similar to solenolide C [18], which were also isolated from the same target organism *Briareum stechei*, and its absolute configuration was determined by single-crystal X-ray diffraction analysis in a later study [19]. Based on the biogenetic grounds, briaranes **1**–**4** can be assumed the same absolute configurations as those of solenolide C; therefore, the absolute configurations of **1**–**4** were established as (1*R*,2*R*,3*R*,4*R*,7*S*,8*S*,9*S*,10*S*,11*R*,12*S*,14*S*,17*R*); (1*S*,2*S*,6*S*,7*R*,8*R*,9*S*,10*S*,12*S*, 14*S*,17*R*); (1*S*,2*R*,3*S*,4*R*,6*S*,7*R*,8*R*,9*S*,10*S*,11*R*,17*R*); and (1*S*,2*R*,3*R*,4*R*,6*S*,7*R*,8*R*,9*S*,10*S*,11*R*, 17*R*), respectively.

### 2.3. Bioactivty of Isolated Briaranes

**Figure 4:**
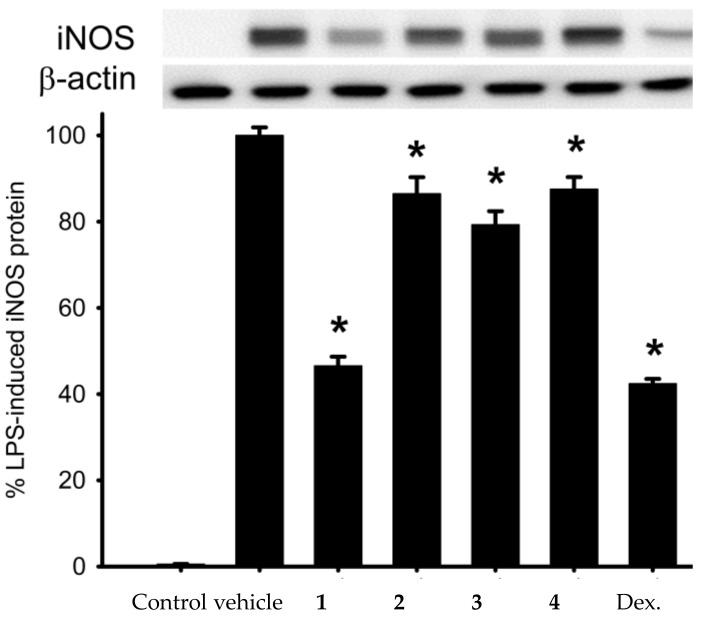
Western blotting showed that briaexcavatolide P (**1**) reduced the expression of iNOS. Data were normalized to the cells treated with LPS only, and cells treated dexamethasone (Dex.) were used as positive control. Data are expressed as the mean ± SEM (*n* = 3). * Significantly different from cells treated with LPS (*p* < 0.05).

It has been well documented that the microbial LPS can activate toll-like receptor-4 (TLR-4) located in mammal cell membrane surface, triggering inflammatory responses through the activation of intracellular signal transduction and the upregulation of pro-inflammatory protein iNOS [20]. Therefore, the determination of the inhibited rate of pro- inflammatory protein iNOS expression in LPS-stimulated macrophage cells can be used as an in vitro screening model for anti-inflammatory compounds [21,22,23]. The anti- inflammatory effect related to the release of iNOS from LPS-stimulated RAW 264.7 macrophage cells by briaranes **1**–**4** was assessed. In a concentration of 10 μM, briaexcavatolide P (**1**) reduced the release of iNOS (46.53%) as compared to results of the vehicle group, which did not, while briaranes **2**–**4** slightly reduced iNOS (Table 2 and Figure 4). These findings seem to be consistent with the results in the literature that demonstrated most briarane-type natural products can potentially be claimed to be anti-inflammatory agents [12]. Structure–activity relationships between **3** and **4** showed that the functional groups at C-2 might did not affect their activities.

## 3. Materials and Methods

### 3.1. General Experimental Procedures

A digital polarimeter (model P-1010; Jasco Corp., Tokyo, Japan) was used to determine the optical rotations of the samples. IR spectra were collected using a spectrophotometer (model Nicolet iS5 FT-IR; Thermo Fisher Scientific, Waltham, MA, USA). ^1^H and ^13^C NMR spectra were recorded on ECZ-400 spectrometer (Jeol Ltd., Tokyo, Japan) for solutions in acetone-*d*_6_ or CDCl_3_ (with residual acetone (δ_H_ 2.04 ppm) and acetone-*d*_6_ (δ_C_ 206.7, 29.8 ppm) or with residual CHCl_3_ (δ_H_ 7.26 ppm) and CDCl_3_ (δ_C_ 77.0 ppm), as internal standards). For coupling constants (*J*), the results are given in frequency units, Hz. For positive mode ESIMS and HRESIMS, the results were obtained using a SolariX FTMS mass spectrometer (7 Tesla; Bruker, Bremen, Germany). The extracted samples were separated by column chromatography with silica gel (range, 230−400 mesh; Merck, Darmstadt, Germany). Thin-layer chromatography plates with silica gel coated with fluorescent indicator F_254_ were employed. For visualization, the plates were charred with 10% (*v*/*v*) aqueous sulfuric acid solution, then heated at 105 °C until spots were observed. For normal-phase HPLC (NP-HPLC) separation, a system containing a pump (Hitachi model L-7110; Tokyo, Japan) and an injection interface (No. 7725i; Rheodyne, Rohnert Park, CA, USA) was employed, which was equipped with a silica preparative column with a dimension of 250 × 20 mm and a 5 μm particle size (YMC-Pack SIL; Sigma-Aldrich, St. Louis, MO, USA). For reverse-phase HPLC (RP-HPLC) separation, a system composed of a pump (model L-2130, Hitachi, Tokyo, Japan) and a diode-array detector (model L-2455, Hitachi, Tokyo, Japan) was used, which was equipped with a C18 preparative column with a dimension of 250 × 21 mm and a 5 μm particle size (Luna, C18(2) 100Å, AXIA; Phenomenex, Torrance, CA, USA).

### 3.2. Animal Material

The specimens of Formosan *Briareum stechei* used for this study were collected from Orchid Island, Taitung, Taiwan in 2017. A voucher specimen was deposited in the National Museum of Marine Biology and Aquarium (NMMBA), Taiwan (NMMBA-TW-SC-2017-418). The specimens of Okinawan *B. stechei* were collected in the Ie Island, Okinawa, Japan in 2019. A voucher specimen was deposited in the NMMBA, Taiwan (NMMBA-JP-SC-2019-001). These two samples were identified based on their morphology and micrographs of the coral sclerites using comparison as described in a previous study [8].

### 3.3. Extraction and Isolation

#### 3.3.1. Formosan *Briareum stechei*

Sliced bodies (wet/dry weight = 1344/568 g) of the specimen were extracted with supercritical CO_2_ to provide an extract (58.9 g). Partial extract (22.5 g) was then applied to a silica gel column chromatography (Si C.C.) and eluted with gradients of *n*-hexane/ethyl acetate (EtOAc) to furnish fractions A–K. Fraction F was purified by NP-HPLC using a mixture of *n*-hexane/acetone (4:1) to yield sub-fractions F1–F13. Fraction F6 was repurified by RP-HPLC, using a mixture of methanol (MeOH)/H_2_O (65:35; at a flow rate = 5 mL/min) to yield briaexcavatolide P (**1**) (0.8 mg).

Briaexcavatolide P (**1**): colorless prisms; ${\left[\alpha \right]}_{D}^{25}$ + 223 (c 0.04, CHCl_3_) (reference [15], ${\left[\alpha \right]}_{D}^{27}$ + 167 (c 1.0, CHCl_3_)); IR (ATR) ν_max_ 3488, 1783, 1731 cm^−1^; the ^1^H and ^13^C NMR data of **1** are in full agreement with those reported previously [15]; ESIMS: *m*/*z* 633 [M + Na]^+^.

#### 3.3.2. Okinawan *Briareum stechei*

Freeze-dried and sliced bodies (wet/dry weight = 618/305 g) of the coral specimen were extracted with a 1:1 mixture of MeOH and dichloromethane (CH_2_Cl_2_) to give 42.7 g of crude extract, which was then subjected to liquid–liquid partition between EtOAc and H_2_O. The EtOAc phase (15.1 g) was applied on Si C.C. and eluted with a gradient solvent system of *n*-hexane/EtOAc mixtures (100% *n*-hexane−100% EtOAc, stepwise) to obtain 11 subfractions A–K. Fraction F was further subjected to the NP-HPLC with a solvent system of *n*-hexane/EtOAc mixture (3:2; flow rate = 5 mL/min) to yield 10 subfractions F1–F10. Fraction F8 was purified by the RP-HPLC using an isocratic solvent system of MeOH/H_2_O mixture (60:40; flow rate = 5 mL/min) to afford **4** (0.3 mg). Fraction G was subjected to the NP-HPLC with a mixture of *n*-hexane/acetone (3:1; flow rate = 5 mL/min) to yield 10 subfractions G1–G10. Fraction G7 was further purified by the RP-HPLC using an isocratic solvent system of MeOH/H_2_O mixture (60:40; flow rate = 5 mL/min) to afford **2** (0.4 mg) and **3** (0.2 mg), respectively. 

Briastecholide B (**2**): amorphous powder; ${\left[\alpha \right]}_{D}^{24}$ + 130 (*c* 0.02, CHCl_3_); IR (KBr) ν_max_ 3490, 1774, 1735 cm^−^^1^; ^1^H (400 MHz, acetone-*d*_6_) and ^13^C (100 MHz, acetone-*d*_6_) NMR data, see Table 1; ESIMS: *m/z* 523 [M + Na]^+^, 525 [M + 2 + Na]^+^; HRESIMS: *m/z* 523.1703 (calculated for C_24_H_33_ClO_9_ + Na, 523.1705).

Briastecholide C (**3**): amorphous powder; ${\left[\alpha \right]}_{D}^{21}$ − 73 (*c* 0.01, CHCl_3_); IR (KBr) ν_max_ 3474, 1757, 1735, 1672 cm^−1^; ^1^H (400 MHz, CDCl_3_) and ^13^C (100 MHz, CDCl_3_) NMR data, see Table 1; ESIMS: *m/z* 477 [M + Na]^+^, 479 [M + 2 + Na]^+^; HRESIMS: *m/z* 477.1284 (calculated for C_22_H_27_ClO_8_ + Na, 477.1287).

Briarenol R (**4**): amorphous powder; ${\left[\alpha \right]}_{D}^{23}$ − 61 (*c* 0.01, CHCl_3_) (ref. [16] ${\left[\alpha \right]}_{D}^{22}$ − 55 (*c* 0.2, CHCl_3_)); IR (KBr) ν_max_ 3452, 1783, 1742, 1680 cm^−1^; ^1^H and ^13^C NMR data of **4** are in full agreement with those reported previously [16]; ESIMS: *m/z* 519 [M + Na]^+^, 521 [M + 2 + Na]^+^.

### 3.4. In Vitro Inflammatory Assay

Pro-inflammatory protein-inducible nitric oxide synthase (iNOS) in macrophages were induced by incubating them for 16 h in a medium containing LPS (0.01 μg/mL) without compounds. For the anti-inflammatory activity assay, compounds or positive control (dexamethasone) were added to the cells 5 min before the lipopolysaccharides (LPS) administrate. After exposure to the compounds or dexamethasone, the macrophages were washed with ice-cold phosphate-buffered saline (PBS), lysed in ice-cold lysis buffer (50 mM Tris, pH 7.5, 150 mM NaCl, 1% Triton X-100, 100 μg/mL phenylmethylsulfonyl fluoride and 1 μg/mL aprotinin) and centrifuged at 20,000× *g* for 30 min at 4 °C. The supernatants were decanted and reserved for Western blotting. Protein concentrations were measured using a protein assay kit (Bio-Rad, Hercules, CA, USA). The method of Western blotting was similar to that in our previous study [24]. Anti-β-actin antibody was obtained from Sigma Chemical (St. Louis, MO, USA). Anti-iNOS antibody was purchased from Cayman Chemical Company (Ann Arbor, MI, USA). Horse radish peroxidase-conjugated secondary antibodies were obtained from Jackson ImmunoResearch Laboratories (West Grove, PA, USA). The images of Western blotting were obtained using the UVP BioChemi Imaging System (UVP, Upland, CA, USA). Relative densitometric quantification of the Western blotting band was performed using LabWorks 4.0 software (UVP LLC, Upland, CA, USA). The intensity of the LPS only group was set at 100%. The β-actin was used as the loading/internal control. 

## 4. Conclusions

In a continuation of our search for briaranes from *B. stechei*, briaexcavatolide P (**1**) found in this study was previously isolated from *B. excavatum* collected in the waters of Taiwan. In addition, two new chlorinated briaranes, briastecholides B (**2**) and C (**3**), together with a known analogue, briarenol R (**4**), were further identified from *B. stechei*. This octocoral is originally flourishing in the waters of Okinawa, where the Kuroshio current and South China Sea surface current converge to provide high biodiversity. Moreover, the structures, especially the absolute configurations of compounds **1**–**4**, were determined based on spectroscopic data and biogenetic consideration. In bioassay, compound **1** displayed moderate activity against LPS-induced iNOS production. Accordingly, the diverse diterpenoids and their potential pharmacological effects of *B. stechei* demonstrated it worthy of further exploration.

## Figures and Tables

**Table 1:** ^1^H and ^13^C NMR data for briaranes **2** and **3** (δ in ppm).

	2		3	
C/H	δ_H_^a^ (*J* in Hz)	δ_C_, ^b^ Type	δ_H_^f^ (*J* in Hz)	δ_C_, ^g^ Type
1		48.7, C		41.6, C
2	5.98 d (8.8)	75.1, CH	3.14 dd (8.8, 3.2)	75.7, CH
3α/β	1.46 m; 3.25 m	29.3, CH_2_	3.43 dd (8.8, 4.0)	62.4, CH
4α/β	2.41 m; 2.36 m	34.2, CH_2_	3.71 d (4.0)	58.3, CH
5		148.9, C		134.5, C
6	5.21 d (4.0)	56.3, CH ^c^	5.35 ddd (2.8, 2.8, 2.8)	60.9, CH
7	5.00 br s	83.8, CH	5.06 d (2.8)	76.3, CH
8		83.9, C		84.3, C
9	4.88 d (7.6)	79.2, CH ^c^	5.54 d (8.4)	68.4, CH
10	3.16 s	44.0, CH	2.34 dd (8.4, 4.0)	39.2, CH
11		155.7, C	2.82 qd (7.6, 4.0)	44.9, CH
12	4.14 dd (11.6, 6.0)	69.8, CH		202.5, C
13α/β	2.04 m; 1.58 ddd (14.4, 11.6, 3.2)	37.9, CH_2_	5.86 d (10.0)	123.1, CH
14	4.80 dd (3.2, 3.2)	75.8, CH	7.14 d (10.0)	155.8, CH
15	1.06 s	14.9, CH_3_	1.20 s	14.7, CH_3_
16a/b	5.56 br s; 5.26 br s	119.1, CH_2_	5.95 d (2.8); 5.47 d (2.8)	118.9, CH_2_
17	2.87 q (6.8)	52.1, CH	2.48 q (6.8)	45.5, CH
18	1.01 d (6.8)	6.6, CH_3_	1.23 d (6.8)	6.2, CH_3_
19		175.9, C ^c^		173.8, C
20	5.41 br s; 5.19 br s	106.6, CH_2_	1.29 d (7.6)	14.7, CH_3_
OH-2			2.21 d (3.2)	
OH-8	3.76 s		3.43 s	
OH-9	5.52 d (7.6)			
OH-12	4.08 d (6.0)			
OAc-2		170.7, C ^d^		
	1.89 s	21.3, CH_3_^e^		
OAc-9				169.4, C
			2.24 s	21.9, CH_3_
OAc-14		171.2, C ^d^		
	1.89 s	21.5, CH_3_^e^		

^a^ 400 MHz, acetone-*d*_6_. ^b^ 100 MHz, acetone-*d*_6_. ^c^ The ^13^C chemical shifts were assigned by the assistance of HSQC and HMBC spectra. ^d,e^ Data exchangeable. ^f^ 400 MHz, CDCl_3_. ^g^ 100 MHz, CDCl_3_.

**Table 2:** Effects of briaranes **1**–**4** on LPS-induced pro-inflammatory iNOS protein expression in macrophages.

Compound (10 µM)	iNOS
	Expression (% of LPS)
Control	0.51 ± 0.09
Vehicle	100.00 ± 1.87
Briaexcavatolide P (**1**)	46.53 ± 2.15
Briastecholide B (**2**)	86.45 ± 3.85
Briastecholide C (**3**)	79.30 ± 3.13
Briarenol R (**4**)	87.52 ± 2.84
Dexamethasone	42.40 ± 1.11

Data were normalized to those of cells treated with LPS alone, and cells treated with dexamethasone were used as a positive control. Data are expressed as the mean ± SEM (*n* = 3).

## Data Availability

The data presented in this study are available on request from the corresponding author.

## Supplementary Materials

The following are available online, Figure S1: ESIMS spectrum of **2**, Figure S2: HRESIMS spectrum of **2**, Figure S3: IR spectrum of **2**, Figure S4: ^1^H NMR spectrum of **2** in acetone-*d*_6_ at 400 MHz, Figure S5: ^13^C NMR spectrum of **2** in acetone-*d*_6_ at 100 MHz, Figure S6: HSQC spectrum of **2**, Figure S7: HMBC spectrum of **2**, Figure S8: ^1^H‒^1^H COSY spectrum of **2**, Figure S9: NOESY spectrum of **2**, Figure S10: ESIMS spectrum of **3,** Figure S11: HRESIMS spectrum of **3**, Figure S12: IR spectrum of **3**, Figure S13: ^1^H NMR spectrum of **3** in CDCl_3_ at 400 MHz, Figure S14: ^13^C NMR spectrum of **3** in CDCl_3_ at 100 MHz, Figure S15: HSQC spectrum of **3**, Figure S16: HMBC spectrum of **3**, Figure S17: ^1^H‒^1^H COSY spectrum of **3**, Figure S18: NOESY spectrum of **3**.

## Sample Availability

Samples of the compounds **1**–**4** are not available from the authors.
