# Supplementary material for: Briarane-Related Diterpenoids from Octocoral Briareum stechei
Source: Molecules. 2021 Nov 13;26(22):6861. doi: 10.3390/molecules26226861 (PMC8619598; doi:10.3390/molecules26226861)
Supplement: Supplementary file 1 [file molecules-26-06861-s001.zip › molecules-1439637-supplementary.pdf]

Supplementary materials

Figure S1. ESIMS spectrum of compound **2**....2
Figure S2. HRESIMS spectrum of compound **2**....3
Figure S3. IR spectrum of compound **2**....3
Figure S4. ^{1}H NMR spectrum (400 MHz) of compound **2** in acetone-d_6....4
Figure S5. ^{13}C NMR spectrum (100 MHz) of compound **2** in acetone-d_6....4
Figure S6. HSQC spectrum of compound **2** in acetone-d_6....5
Figure S7. HMBC spectrum of compound **2** in acetone-d_6....5
Figure S8. ^{1}H–^{1}H COSY spectrum of compound **2** in acetone-d_6....6
Figure S9. NOESY spectrum of compound **2** in acetone-d_6....6
Figure S10. ESIMS spectrum of compound **3**....7
Figure S11. HRESIMS spectrum of compound **3**....8
Figure S12. IR spectrum of compound **3**....8
Figure S13. ^{1}H NMR spectrum (400 MHz) of compound **3** in $CDCl_3$....9
Figure S14. ^{13}C NMR spectrum (100 MHz) of compound **3** in $CDCl_3$....9
Figure S15. HSQC spectrum of compound **3** in $CDCl_3$....10
Figure S16. HMBC spectrum of compound **3** in $CDCl_3$....10
Figure S17. ^{1}H–^{1}H COSY spectrum of compound **3** in $CDCl_3$....11
Figure S18. NOESY spectrum of compound **3** in $CDCl_3$....11

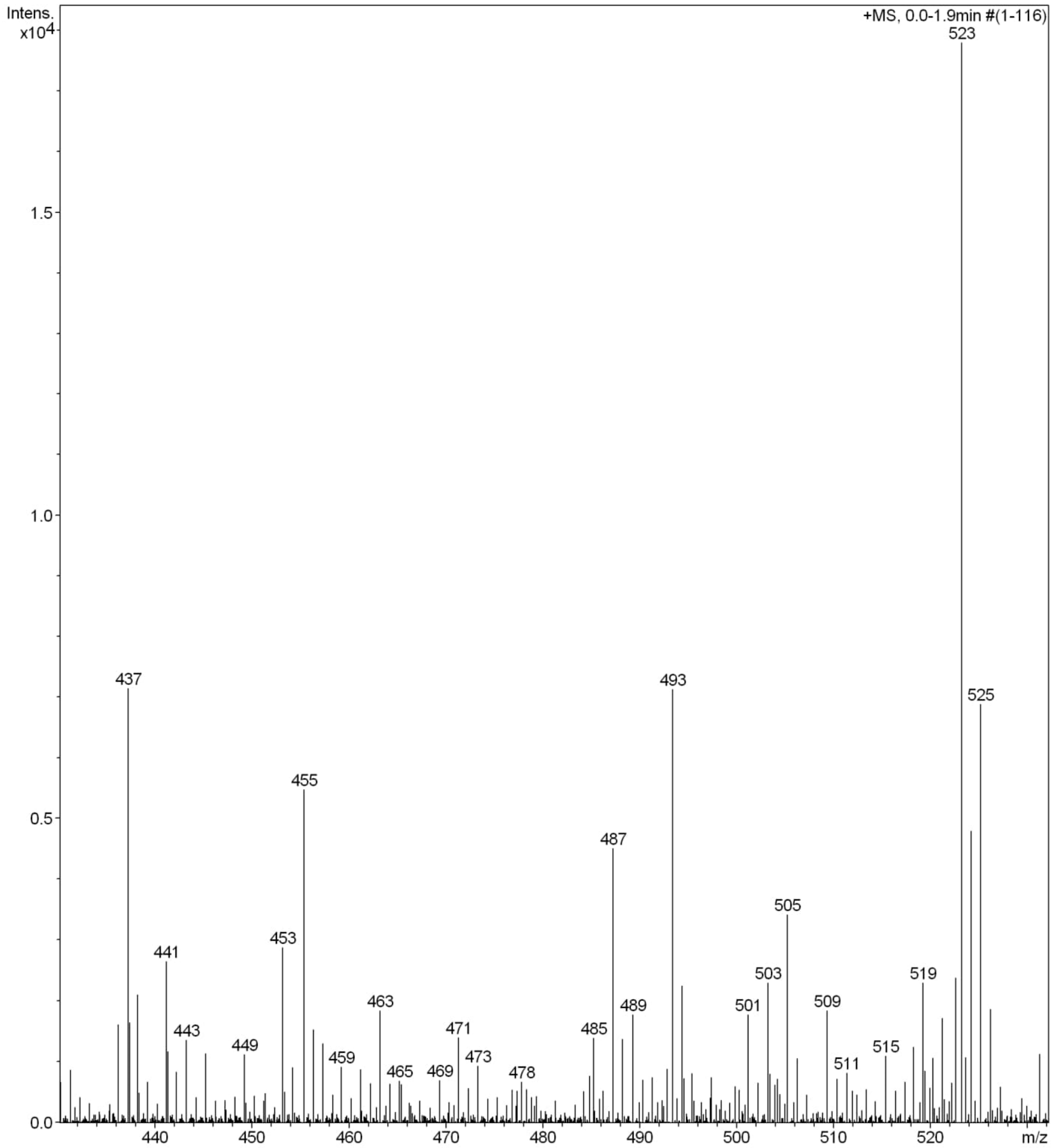

Figure S1. ESIMS spectrum of compound **2**

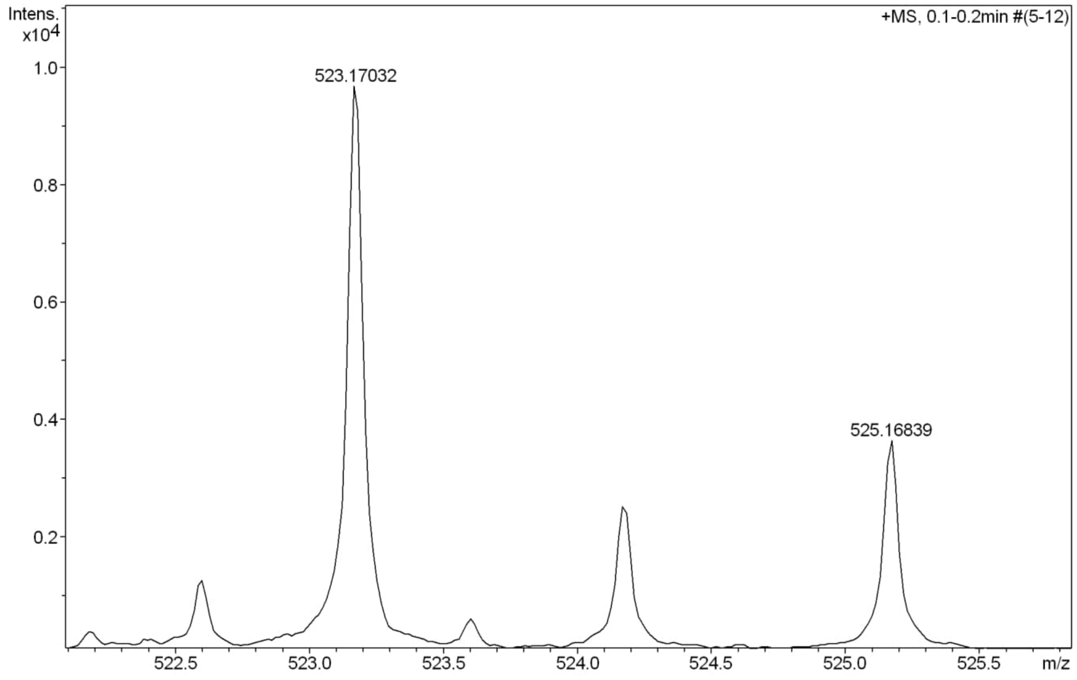

Meas. m/z	#	Formula	Score	m/z	err [mDa]	err [ppm]	mSigma	rdb	e⁻ Conf	N-Rule
523.17032	1	C 24 H 33 Cl Na O 9	100.00	523.17053	0.21	0.40	10.9	7.5	even	ok

Figure S2. HRESIMS spectrum of compound **2**

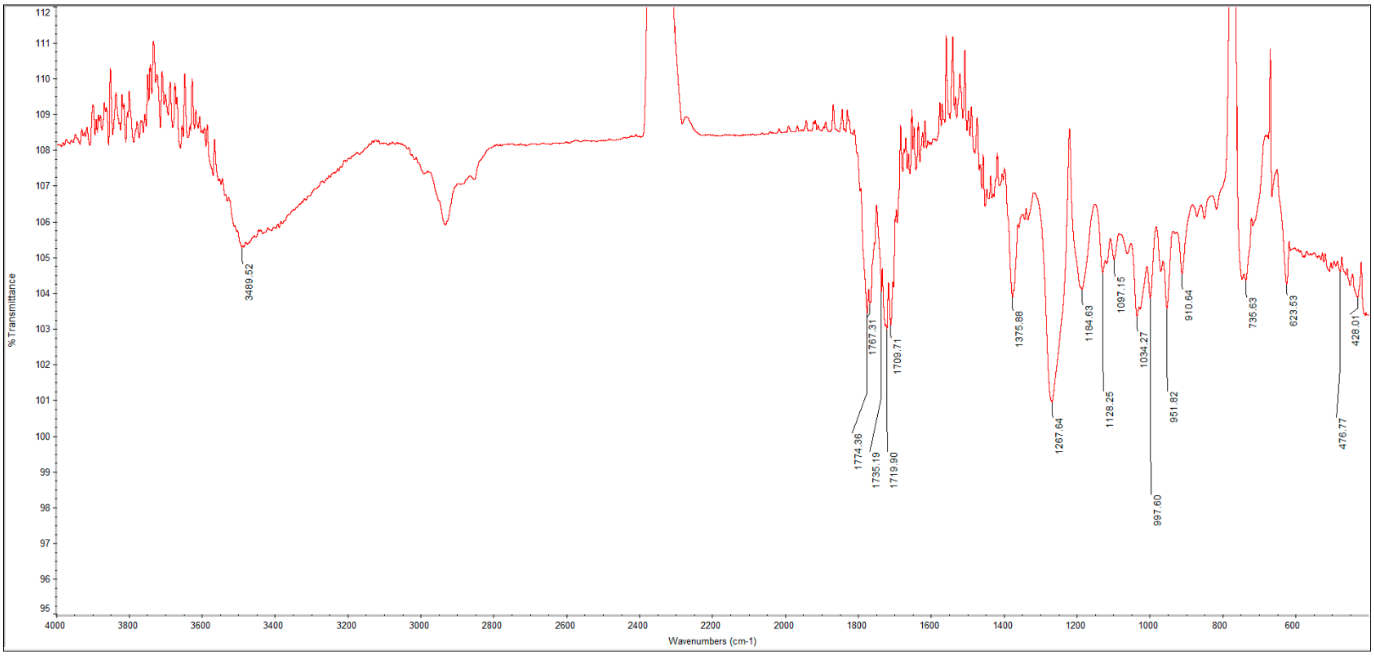

Figure S3. IR spectrum of compound **2**

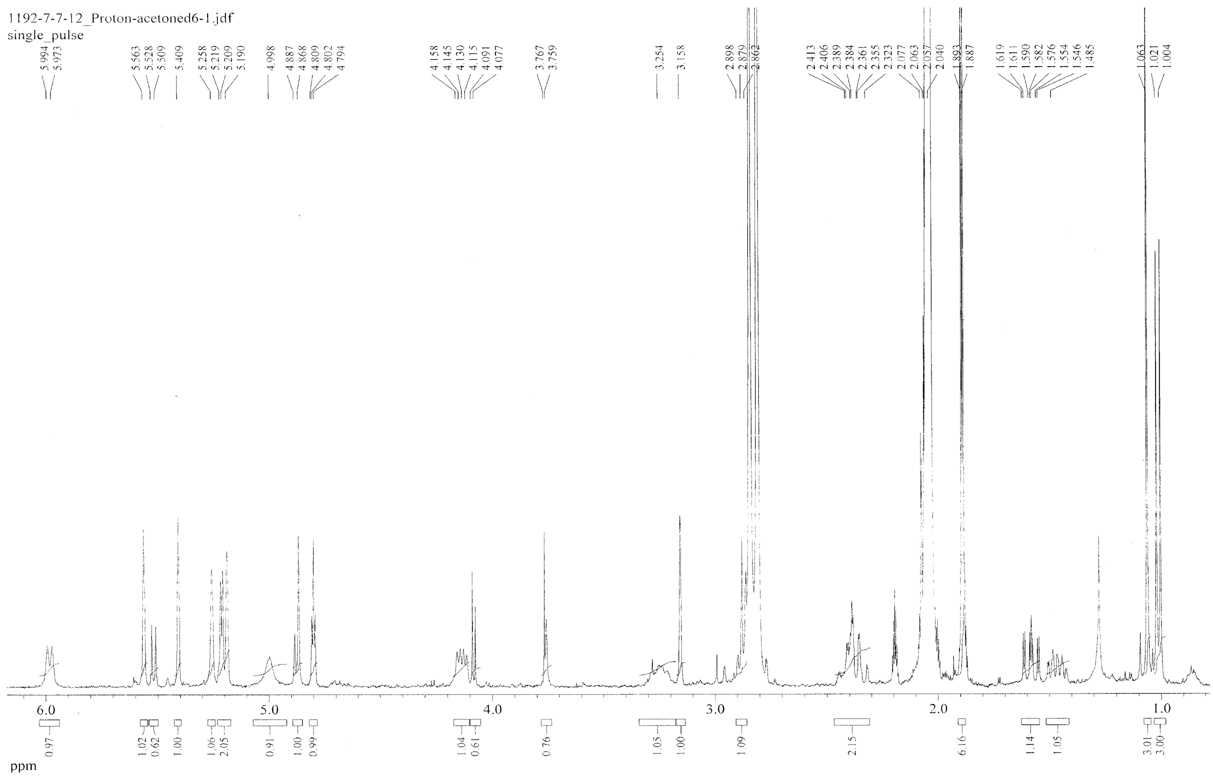

Figure S4. ^{1}H NMR spectrum (400 MHz) of compound **2** in acetone-d_6

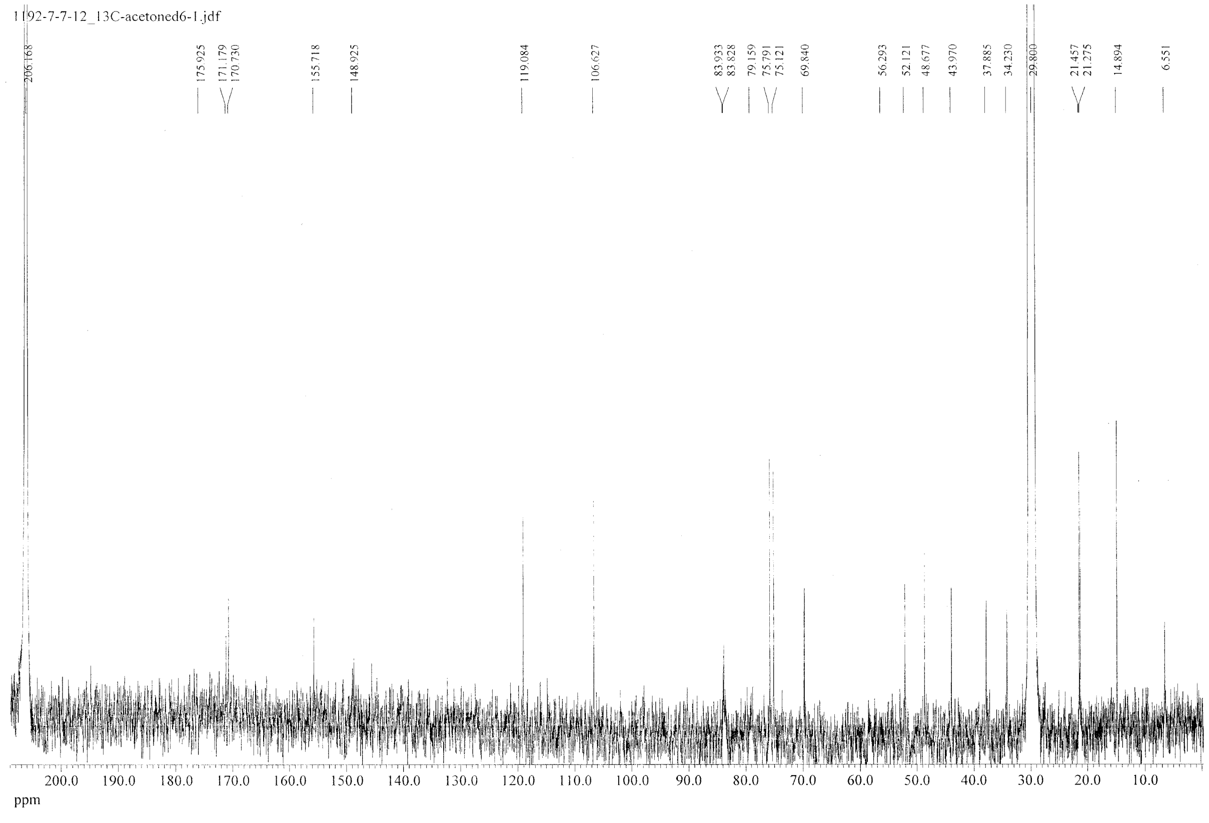

Figure S5. ^{13}C NMR spectrum (100 MHz) of compound **2** in acetone-d_6

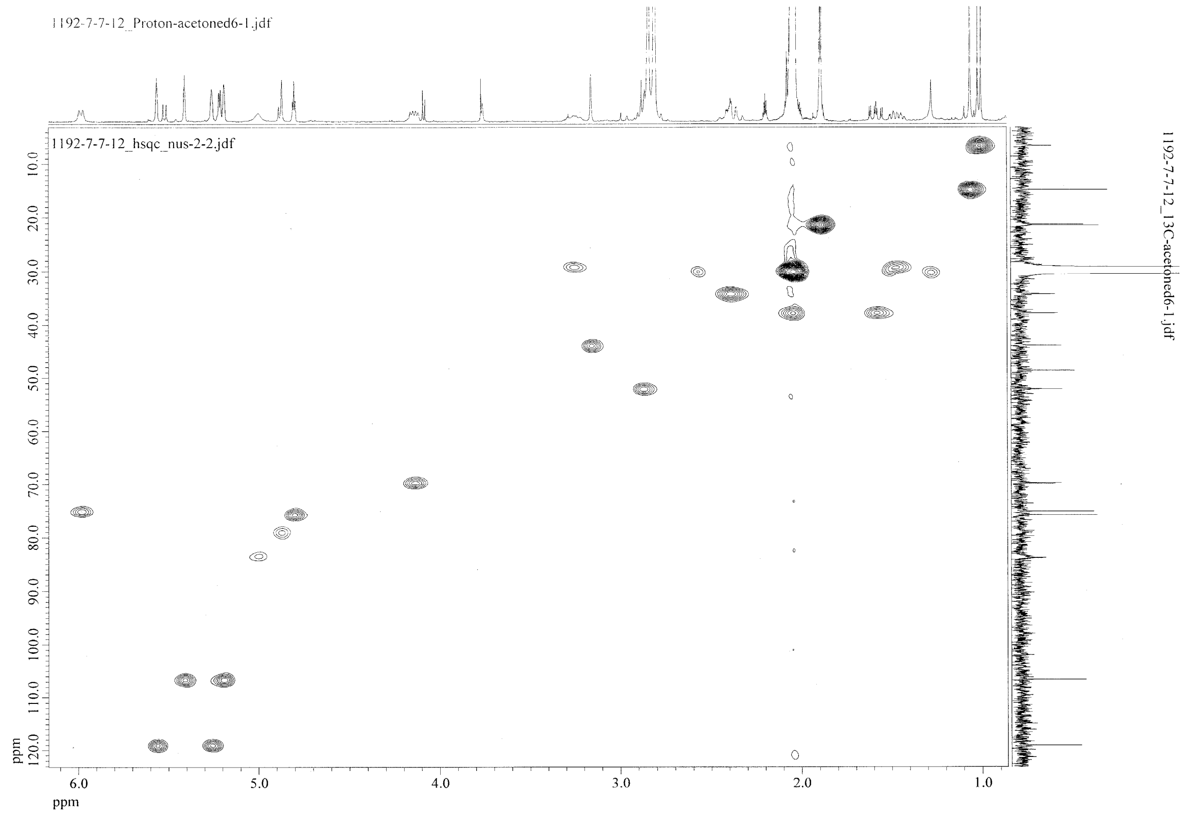

Figure S6. HSQC spectrum of compound **2** in acetone-d_6

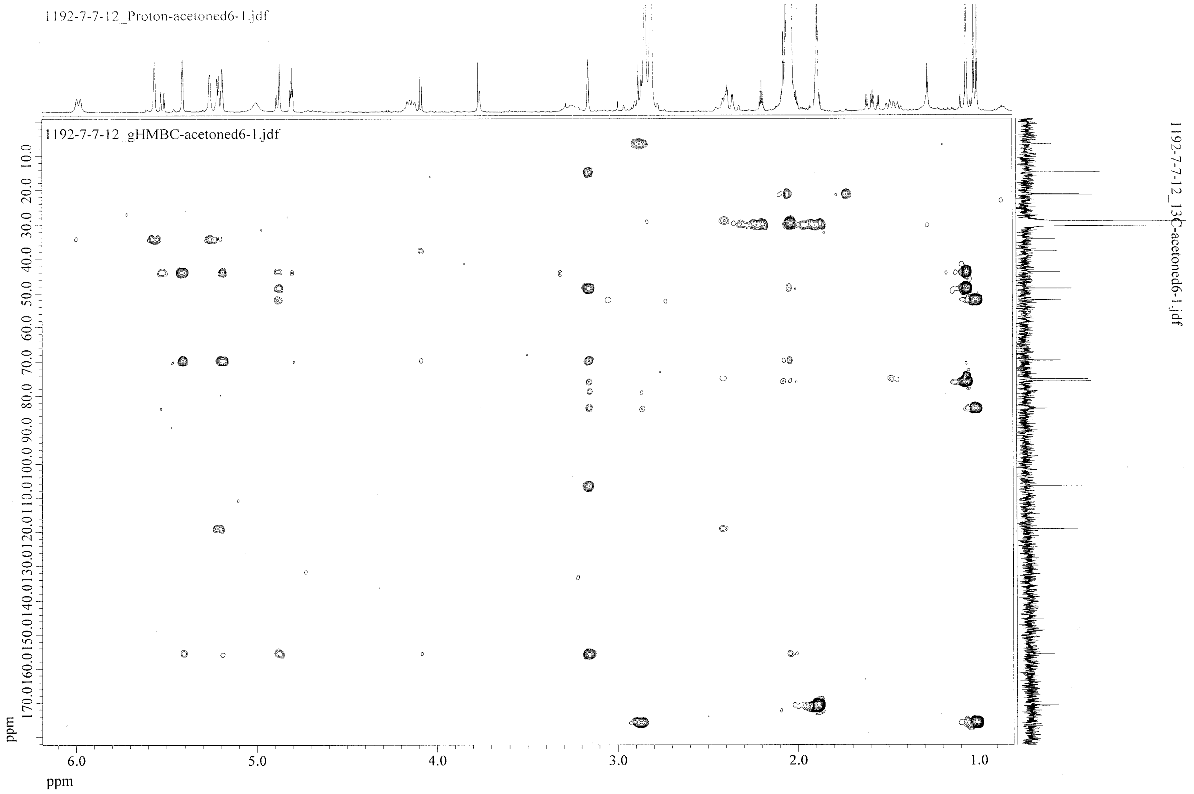

Figure S7. HMBC spectrum of compound **2** in acetone-d_6

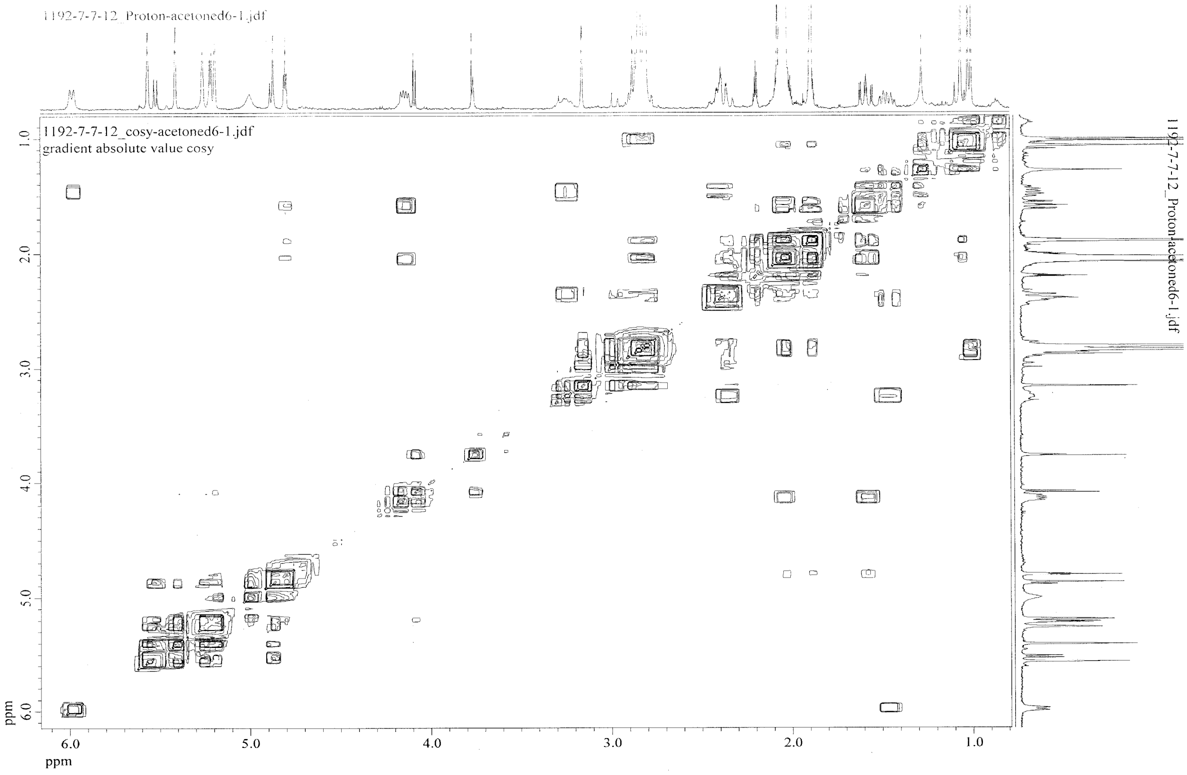

Figure S8. ^{1}H–^{1}H COSY spectrum of compound **2** in acetone-d_6

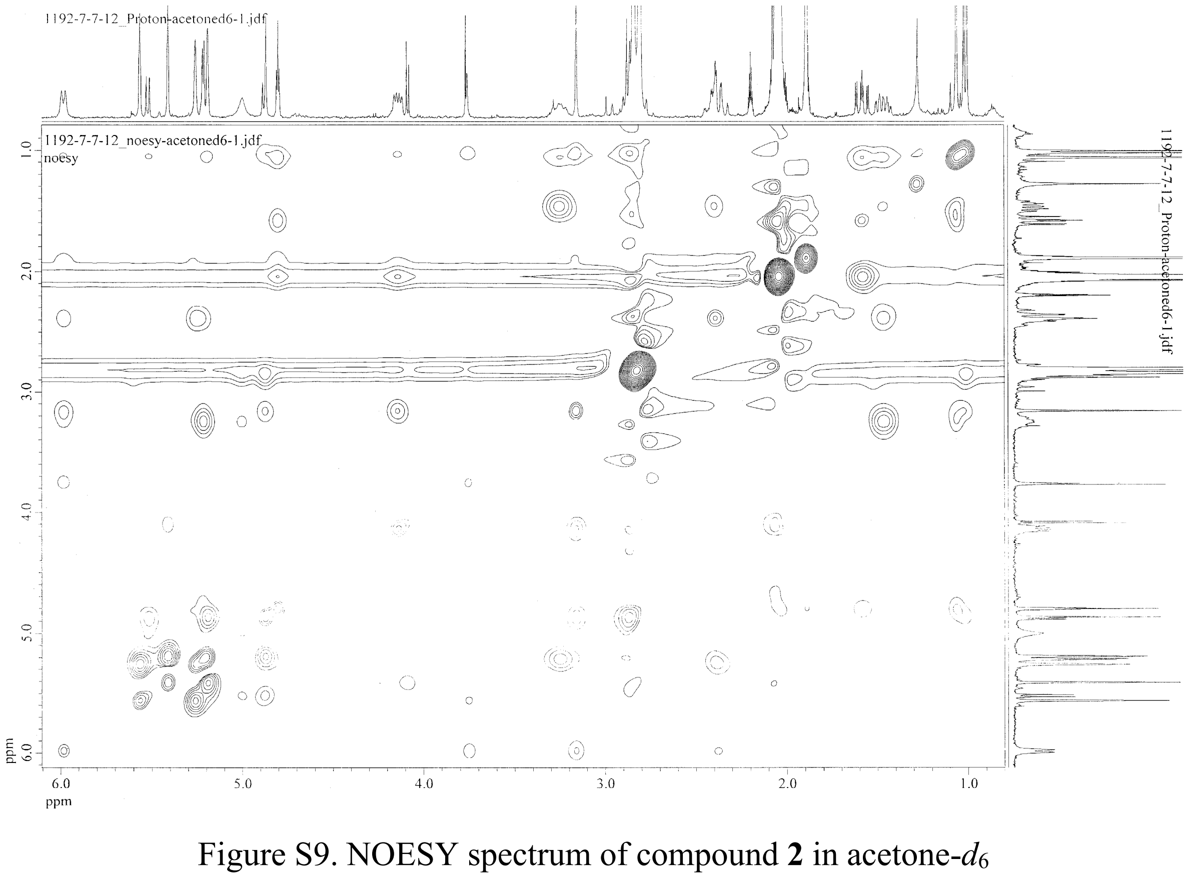

Figure S9. NOESY spectrum of compound **2** in acetone-d_6

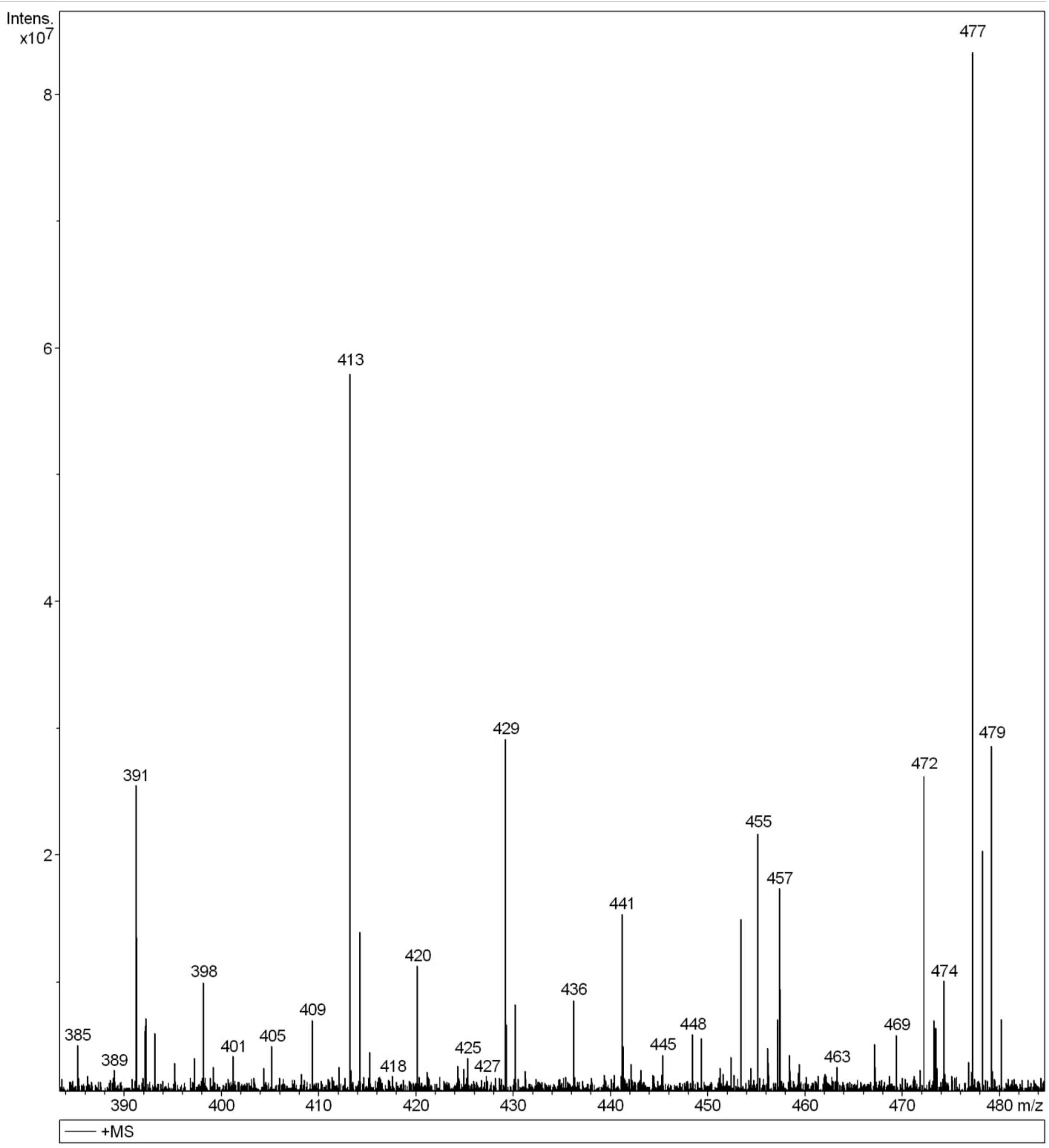

Figure S10. ESIMS spectrum of compound **3**

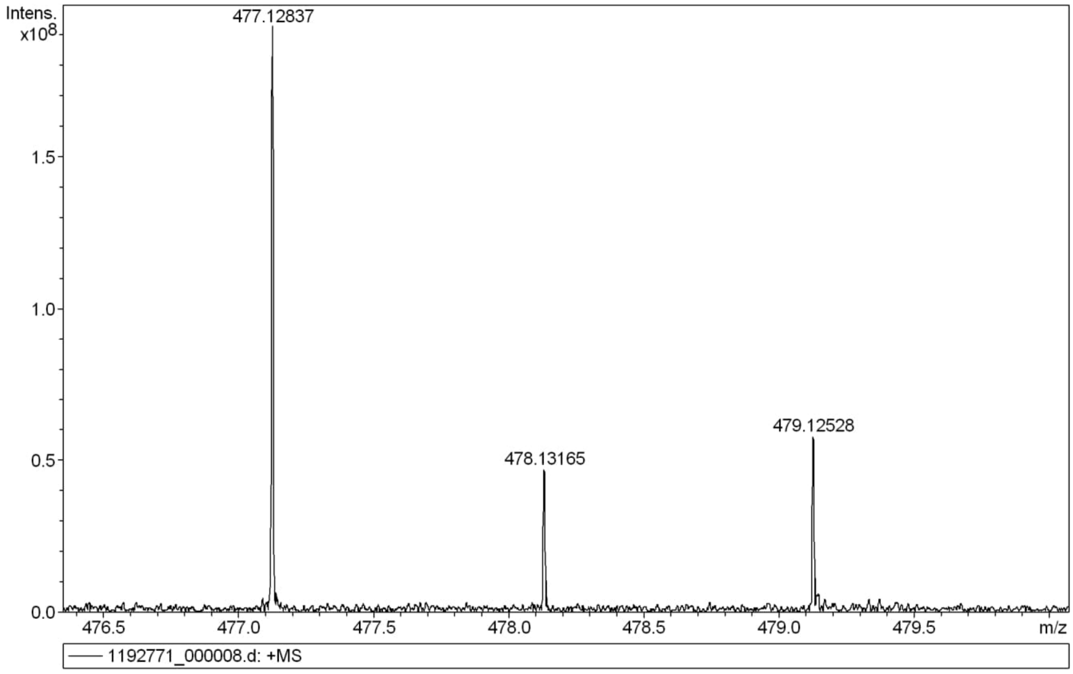

Meas. m/z	#	Formula	Score	m/z	err [mDa]	err [ppm]	mSigma	rdb	e⁻ Conf	N-Rule
477.12837	1	C 22 H 27 Cl Na O 8	100.00	477.12867	0.30	0.62	29.9	8.5	even	ok

Figure S11. HRESIMS spectrum of compound **3**

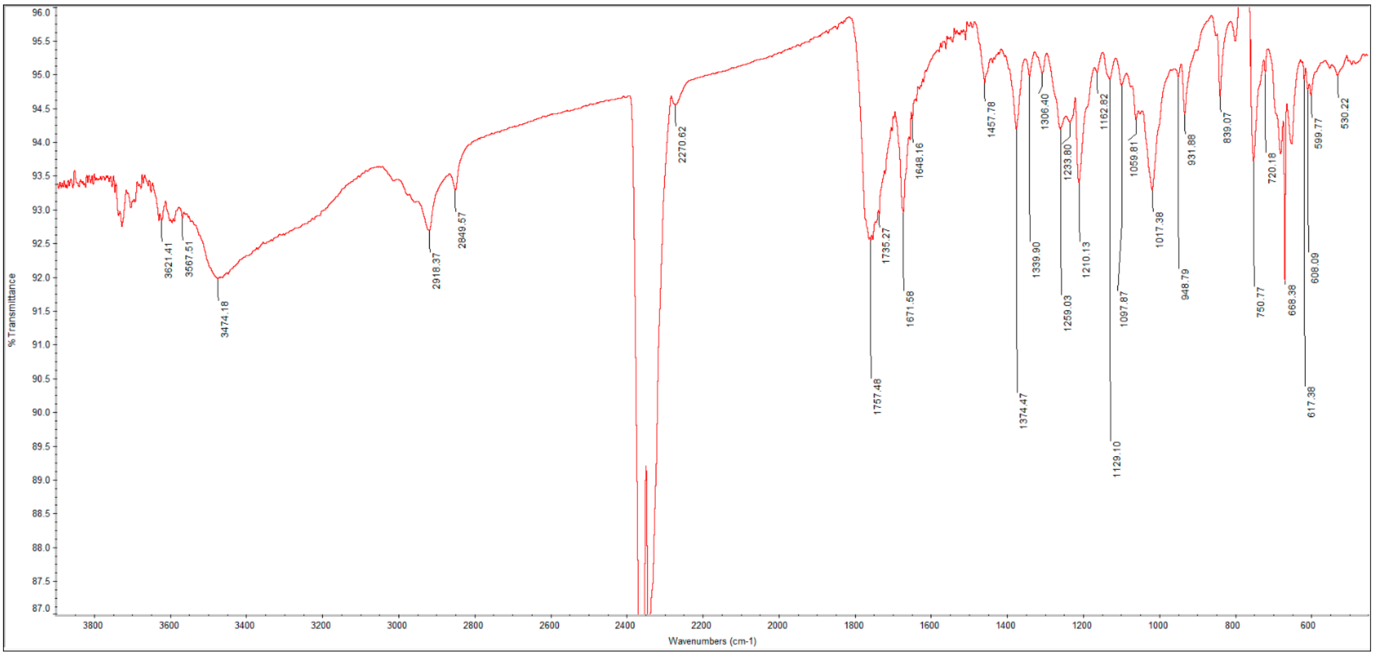

Figure S12. IR spectrum of compound **3**

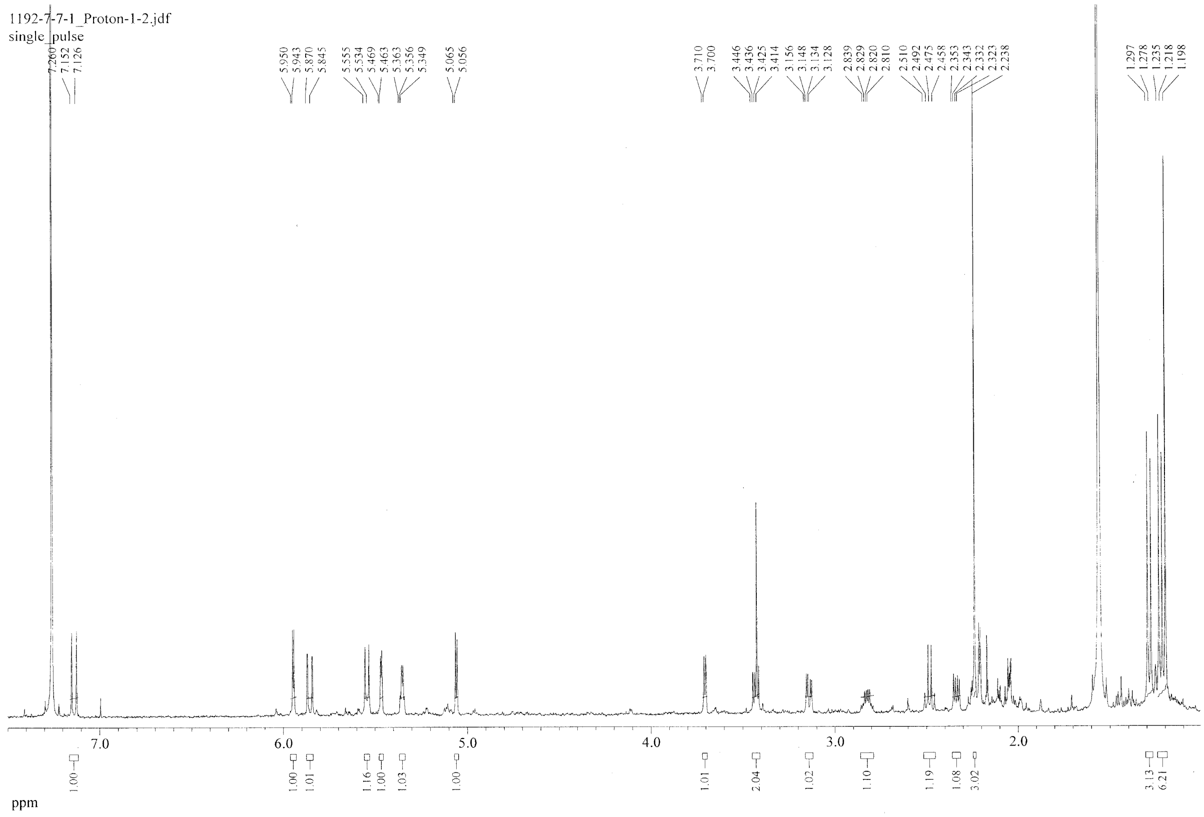

Figure S13. ^{1}H NMR spectrum (400 MHz) of compound **3** in $CDCl_3$

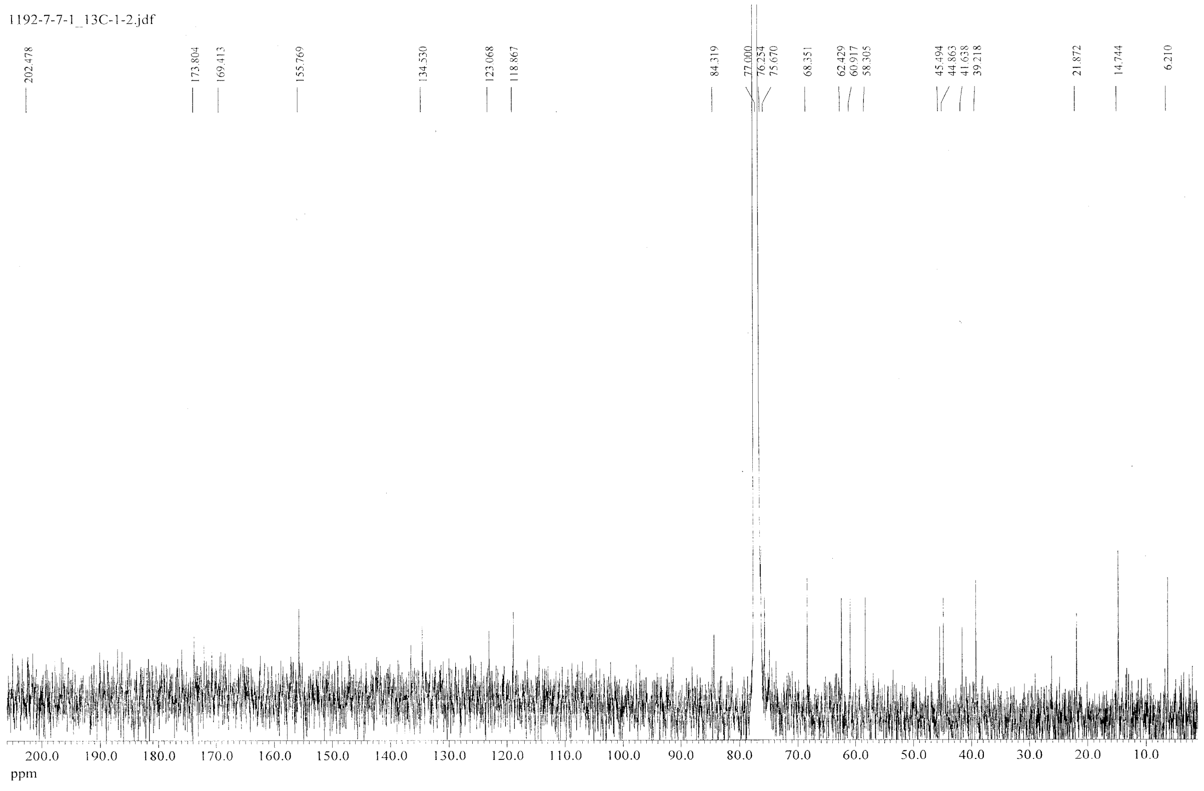

Figure S14. ^{13}C NMR spectrum (100 MHz) of compound **3** in $CDCl_3$

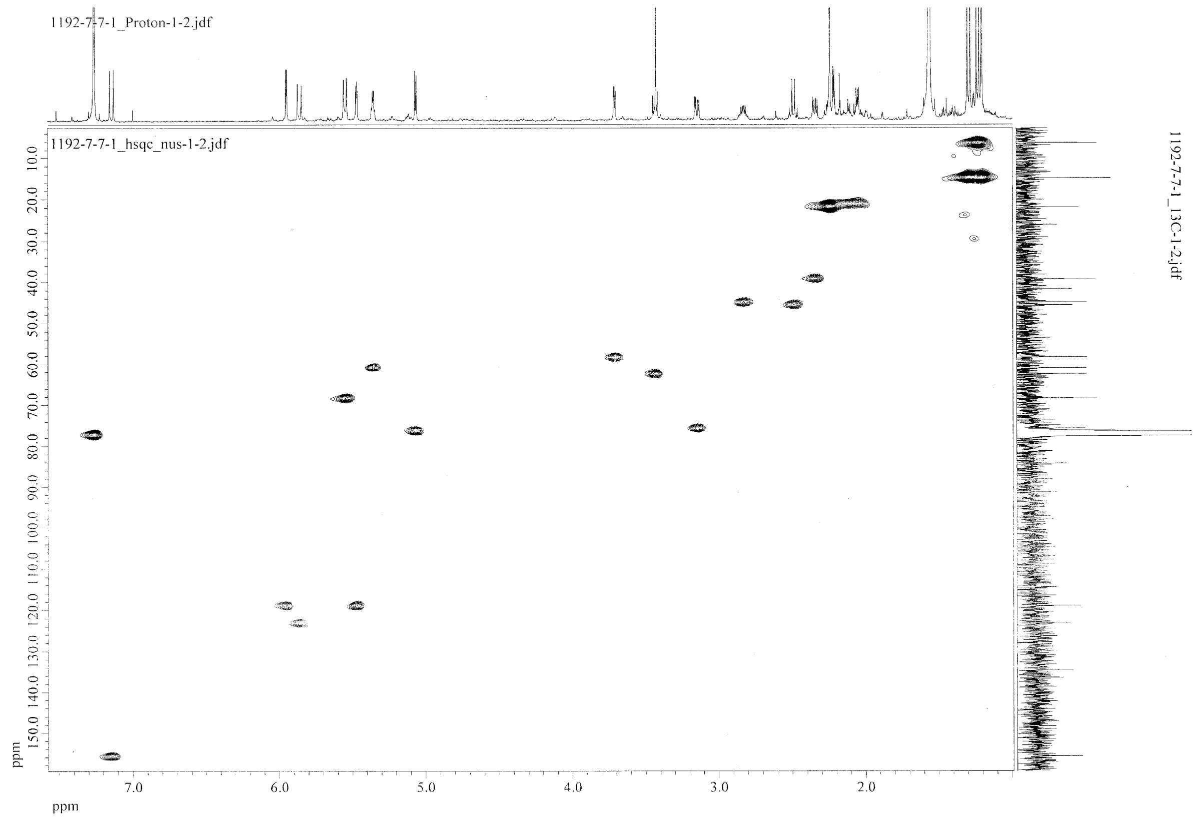

Figure S15. HSQC spectrum of compound **3** in $CDCl_3$

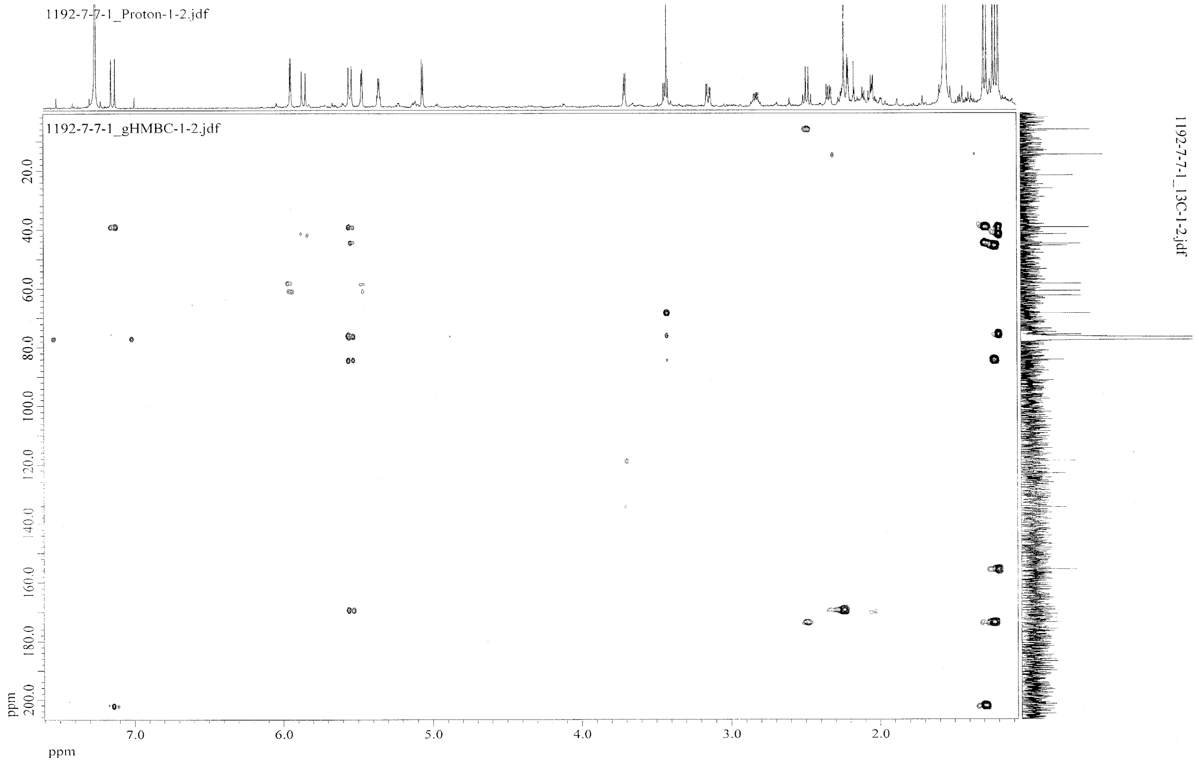

Figure S16. HMBC spectrum of compound **3** in $CDCl_3$

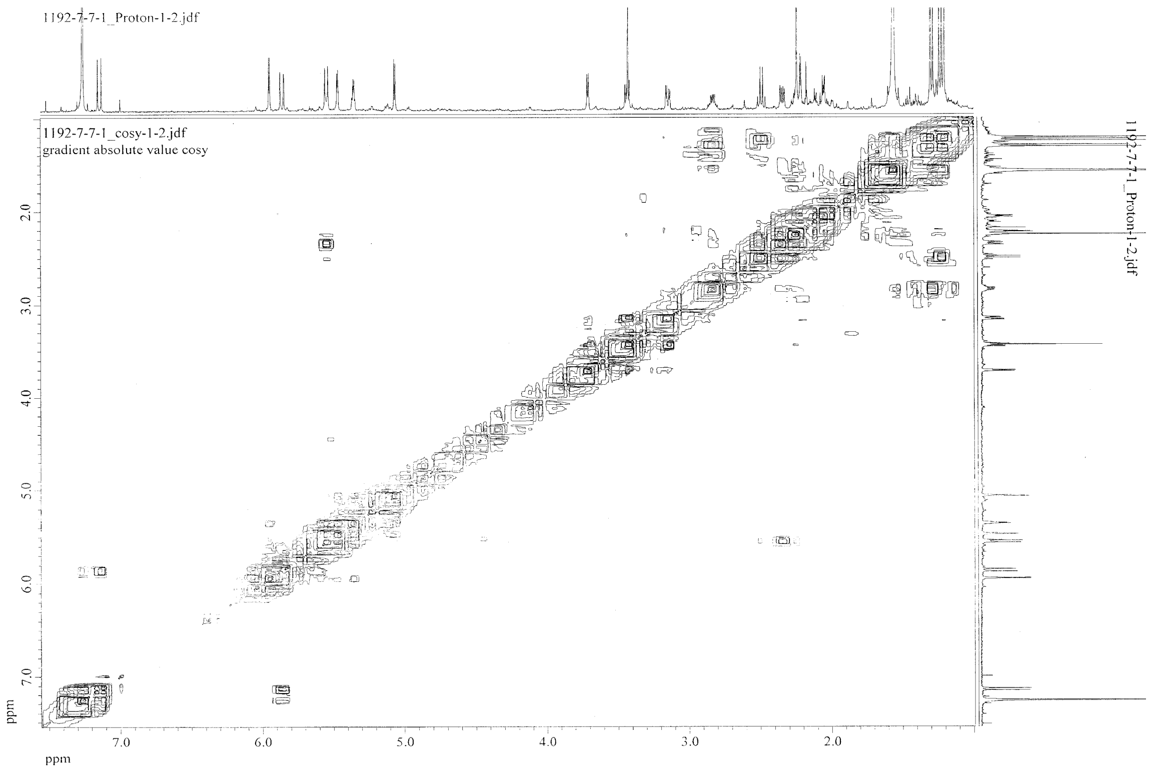

Figure S17. ^{1}H–^{1}H COSY spectrum of compound **3** in $CDCl_3$

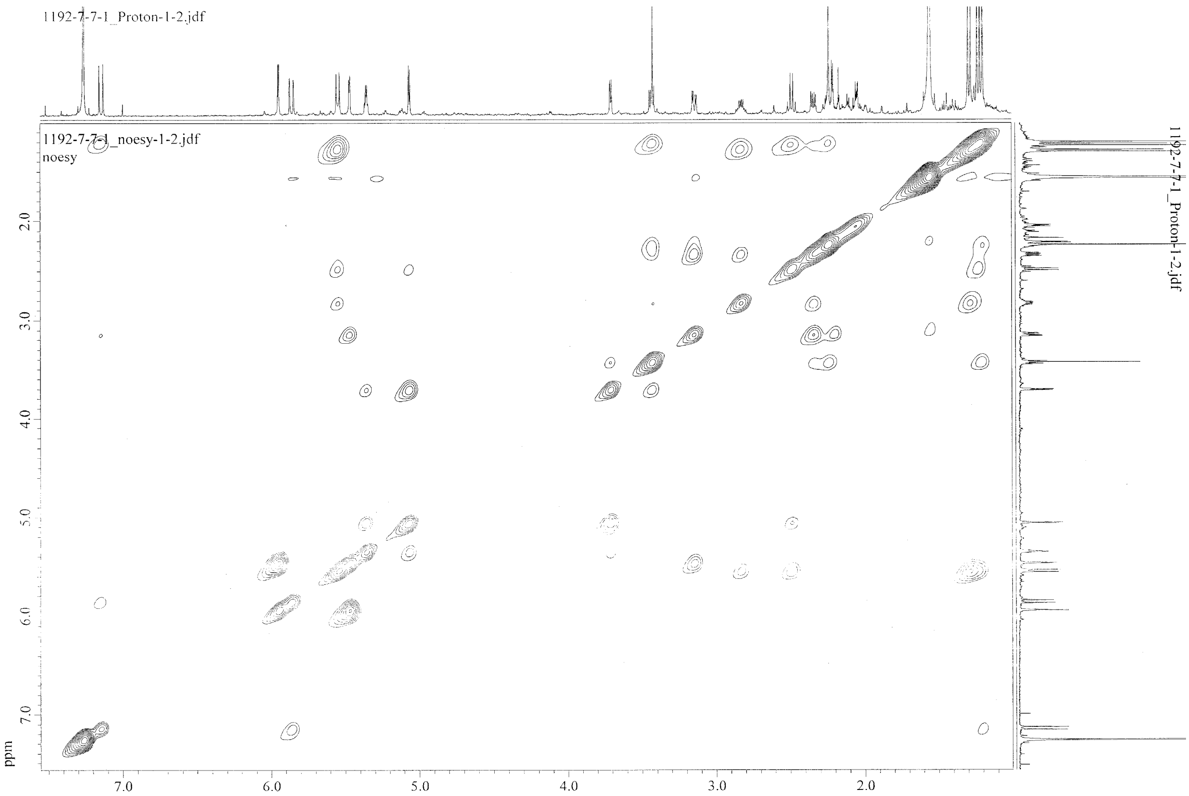

Figure S18. NOESY spectrum of compound **3** in $CDCl_3$
